# Factors affecting institutionalized older peoples’ self-perceived dry mouth

**DOI:** 10.1007/s11136-014-0792-7

**Published:** 2014-08-24

**Authors:** Ying-Chia Huang, Chiao-Lee Chu, Ching-Sung Ho, Shou-Jen Lan, Wen-Yi Chen, Yia-Wung Liang, Yen-Ping Hsieh

**Affiliations:** 1 Department of Healthcare Administration, Asia University, Taichung City, Taiwan, ROC; 2 Department of Long-term Care, National Quemoy University, Kinmen, Taiwan, ROC; 3 Department of Senior Citizen Service Management, National Taichung University of Science and Technology, 129, Sec. 3, Sanmin Rd., Taichung City, 404 Taiwan, ROC

**Keywords:** Long-term care facilities, Dry mouth, Ability to chew food, Older people

## Abstract

**Purpose:**

The purpose of this study was to determine the factors affecting institutionalized older peoples’ self-perceived dry mouth.

**Methods:**

This cross-sectional study was conducted on elderly residents at 22 long-term care facilities. A total of 165 questionnaires were returned from 13 senior citizen welfare institutions (SCWIs) and nine nursing homes. Multiple logistic regression analysis was used to analyze the data obtained.

**Results:**

The results showed that the type of long-term care (LTC) facility, regular oral examinations, wearing dentures, and the ability to chew sticky foods affected self-perceived dry mouth. This study determined an association between the type of LTC facility where the participants lived and self-perceived dry mouth.

**Conclusions:**

The results indicated the importance of providing oral care in order to improve and prevent dry mouth among institutionalized older people living in SCWIs who do not undergo regular oral examinations, wear dentures, and have difficulty chewing sticky foods.

## Introduction

Approximately 20–50 % of elderly people have symptoms of dry mouth. Dry mouth and salivary gland dysfunction are subjective feelings, and they tend to lead to higher risks of fever, pneumonia, and increased mortality; they also have a negative effect on the quality of life of elderly people [1–7]. Existing studies have verified that the factors affecting dry mouth among older people include drugs [8–11], chronic diseases, radiotherapy [12, 13], age, and tooth-brushing frequency [8, 10].

The oral care provided by dentists and caregivers to institutionalized older peoples is usually deficient, thereby rendering their oral status poor [14–16]. A systematic review of the research investigating the oral health care of older people in institutions has shown that increasing knowledge regarding oral care and enhancing easy-to-do oral care behavior is an effective strategy for improving older peoples’ oral care [17]. However, most studies have investigated improvements in older peoples’ oral health status from the caregivers’ perspective [17–19], and few have investigated the potential influence of self-care and oral health behavior on dry mouth from the perspective of older people. Few long-term care (LTC) facilities provide consultations with dentists for their residents. Therefore, the self-oral care of elderly people in LTC facilities or the oral care provided by caregivers is more important [8, 9, 20]. Understanding the factors affecting institutionalized older people’s self-perceived dry mouth can help with developing strategies to prevent and improve the oral care of older people living in LTC facilities.

In this study, a cross-sectional investigation was conducted to determine the factors affecting institutionalized older people’s self-perceived dry mouth, including individual characteristics, self-perceived oral factors, oral health self-care behavior, oral health beliefs, and the self-perceived ability to chew food.

## Materials and methods

### Study design

In this study, a cross-sectional questionnaire survey was conducted to investigate the factors affecting self-perceived dry mouth among the residents of LTC facilities in central Taiwan. The questionnaire was conducted from September to November 2010. This study was approved by the Medical Ethics Committee of Asia University (No. 1006011). Letters of consent were obtained from those agreeing to participate in the study. All interviewers were trained prior to conducting the survey.

### Setting and participants

There are two major types of LTC institutions in Taiwan: nursing homes (NHs), which take care of residents with severe disabilities and provide residents with life and medical care, and senior citizen welfare institutions (SCWIs), which take care of residents with moderate/mild disabilities or physical weaknesses and mainly provide life care. Therefore, the medical care resources provided by NHs are more than those provided by SCWIs. The study sites were 22 LTC facilities, including 13 SCWIs and nine NHs in central Taiwan. The inclusion criteria for this study were (a) having lived in an LTC facility for at least 3 months and (b) being able to give informed consent. The exclusion criterion was residents with dementia or cognitive dysfunction. This study performed face-to-face interviews with older people in the LTC facilities. A total of 220 questionnaires were distributed and 165 valid questionnaires were returned, indicating a return rate of 75 %.

### Questionnaire on factors affecting dry mouth

This study intended to fully understand the relevant factors affecting older people’s self-perceived dry mouth symptoms. Therefore, an original questionnaire, developed for this study, was used to collect the data. The questionnaire consisted of five factors. The questionnaire was tested for validity and amended by three experts (including dentists who had volunteered in LTC facilities for over 6 years and nursing professionals working under LTC facility care models) according to each item’s importance. The total score of the scale was 107. This study performed a pre-test on 20 older people living in LTC facilities in August 2010, and the Cronbach’s alpha was 0.54. After the interviews, 165 valid questionnaires were returned. The Cronbach’s alpha of the scale was 0.71, indicating that the reliability was acceptable.

The questionnaire content and scoring method for the five factors are presented below. All the interviewers were trained before the survey.

#### Individual characteristics

This subscale consisted of eight items: the type of LTC facility, the older peoples’ gender, age, education level, tobacco smoking, chewing betel nut, alcohol consumption, and disease. The diseases were mainly those diagnosed by doctors, including hypertension, heart disease, stroke, diabetes, renal failure, and periodontal disease.

#### Self-perceived oral factors

This subscale had ten items in total, with one item relating to whether the participants wore dentures, and nine relating to whether the participants had experienced the following within 3 months: self-perceived dry mouth symptoms, oral bleeding while brushing their teeth, oral bleeding and thermal sensitivity when eating, difficulty chewing, self-perceived halitosis, missing teeth, loose fillings, loose teeth, or a fever. The response options for all 10 items were zero (*no*) or one (*yes*). The total score was 20, and the Cronbach’s alpha was 0.36.

#### Oral health self-care behavior

This subscale consisted of eight items. The first item assessed the number of oral examinations a dentist had performed on the participants over the past year. The other seven items were related to the tooth-brushing and gargling behaviors of older peoples, which included brushing one’s teeth and gargling after waking up in the morning, brushing teeth after meals, gargling after meals, brushing teeth and gargling before sleeping at night, brushing teeth right after taking food, gargling right after taking food, and the habitual use of floss or floss picks. The responses were scored on a five-point Likert-type scale, with scores ranging from one (*never*) to five (*every time*), for a total score of 35 points. The Cronbach’s alpha for this subscale was 0.82.

#### Oral health beliefs

This subscale consisted of four items, including self-perceived oral status, the importance of semi-annual oral examinations, aggressive oral cleaning, and general oral health. A five-point Likert-type scale was used, ranging from one (*highly disagree*) to five (*highly agree*), with a maximum score of 20 points. A Cronbach’s alpha coefficient of 0.61 was obtained for this subscale, indicating moderate reliability.

#### Self-perceived ability to chew food

This subscale had a total of 24 items. The focus of this part of the questionnaire was self-perceived chewing ability [21]. The subscale was developed according to a three-month observation of the most frequently selected foods at Chinese buffet restaurants in Taiwan. The foods were divided into four categories, including eight fruits (juice and non-pickled or overripe/soft fruits), four fresh foods and meats, eight cooked vegetables (mainly scrambled or braised), and four sticky foods. The foods were ranked within each category according to their chewing difficulty, with the first food in each category being the most difficult to chew and the last food the easiest. The maximum score for each category was eight points, resulting in a total maximum score of 32 points. Higher scores indicated better chewing ability. Cronbach’s alpha was 0.88, indicating moderate reliability.

### Statistical analysis

The data were analyzed using SPSS (version 12.0). In addition to descriptive statistical analysis, this study also used multiple logistic regression analysis to determine whether the participants had self-perceptions of dry mouth, which was used as the dependent variable. The independent variables included individual characteristics, self-perceived oral factors, oral health self-care behavior, oral health beliefs, and self-perceived ability to chew food. Odds ratios (OR) and 95 % confidence intervals (95 % CI) were used to determine the effect size.

## Results

The participants’ individual characteristics are shown in Table 1. The average age of the residents living in LTC facilities was 78.7 years (SD: 10.1 years), and 43.6 % of the participants had an education level at or below elementary school. The most common diseases among the participants were hypertension (35.2 %) and heart disease (16.4 %).

**Table 1:** Individual characteristics

Characteristic	*N*	Percentage
*Gender*		
Male	59	35.8
Female	106	64.2
Institutions type		
Nursing home	73	44.2
Senior citizens welfare institutions	92	55.8
Age (years)^a^	78.7 ± 10.1	
*Education level*		
Illiterate	72	43.6
Elementary school	57	34.5
Senior high school or higher	36	21.9
Disease^a^	1.0 ± 1.1	
Hypertension	58	35.2
Heart disease	27	16.4
Stroke	22	13.3
Diabetes	39	23.6
Renal failure	4	2.4
Periodontal disease	2	13.3
*Tobacco smoking*		
No	159	96.4
Yes	6	3.6
*Betel nut chewing*		
No	153	92.7
Yes, but I have quit	12	7.3
*Drinking*		
No	161	97.6
Yes, but I have quit	4	2.4

^a^Mean ± SD

In terms of the participants’ self-perceived oral factors (Table 2), 58.8 % wore dentures and the most common self-perceived symptoms included missing teeth, chewing difficulty, and dry mouth. In terms of oral health, self-care behavior, 80.6 % had not undergone regular oral examinations within the past year. Each participant had undergone 0.4 ± 0.9 oral examinations over the past year. The most frequent behaviors among the participants were brushing teeth and gargling after waking up, gargling after meals, and brushing teeth and gargling before sleeping at night. Most participants did not habitually use floss or use floss picks (Table 2).

**Table 2:** Self-perceived oral factors, oral health self-care behavior, and oral health beliefs

*Self-perceived oral factors characteristic*	*N*	Percentage
Denture wearing	97	58.8
Self-perceived dry mouth symptoms	94	20.7
Oral bleeding during tooth brushing	25	15.2
Feeling soreness and pain in teeth when eating cold and hot foods	32	19.4
Feeling difficulty in chewing during food ingestion	36	21.8
Self-perceived bad breath	31	18.8
Missing teeth	94	57.0
Prosthodontic may easily become loose	7	4.2
Loose teeth	16	9.7
Fever within the last 3 months	7	4.2
*Oral health self-care behavior characteristic*	Mean ± SD	
Regular oral examinations	0.4 ± 0.9	
Brushing teeth and gargling after waking in the morning	4.3 ± 1.5	
Brushing teeth after meals	2.1 ± 1.6	
Gargling after meals	3.3 ± 1.9	
Brushing teeth and gargling before sleep at night	3.3 ± 1.9	
Brushing teeth after eating anything	2.2 ± 1.7	
Gargling after eating anything	3.1 ± 1.8	
Using floss or floss pick	1.7 ± 1.4	
*Oral health beliefs characteristic*	Mean ± SD	
The current oral condition of the residents is good	3.2 ± 1.3	
It is important to undergo oral examinations semi-annually	3.5 ± 1.2	
Need to undergo aggressive oral cleaning	3.8 ± 1.1	
Oral health of residents is important to their health	3.7 ± 1.2	

With regard to the participants’ oral health beliefs, the participants indicated that they needed to undergo aggressive oral cleaning and that oral health was important to their health (Table 2). They also noted that cooked vegetables and fruits were the easiest to chew (Table 3).

**Table 3:** Self-perceived ability to chew food

Characteristic	Mean ± SD
*Fruits* (*non-pickled fruits, overripe/soft fruits, or juice*)	
Sugar cane (not juice)	5.8 ± 2.5
Sliced guava	
Sliced apple or pear	
Sliced orange	
Sliced star fruit or bell fruit	
Sliced melon or tangerine	
Sliced watermelon or pineapple	
Papaya or banana	
*Fresh foods and meats*	
Squid	5.3 ± 2.8
Soy sauce-braised pork ears	
Fried chicken leg or chicken fillet	
Fish (steamed)	
*Cooked vegetables* (*mainly scrambled or braised food*)	
Stir-fried peanut	6.8 ± 2.1
Boiled bamboo shoots or broccoli	
Sliced cucumber or kidney bean	
Water spinach or cabbage	
Pickled lettuce in soy sauce or pickled cucumber in soy sauce	
Sliced sweet pepper	
Steamed sweet potato or taro	
Overcooked tomato	
*Sticky foods*	
Malt syrup nougat	5.6 ± 2.9
Mochi	
Rice cake	
Rice dumpling	

### Factors associated with self-perceived dry mouth

Multiple logistic regression analysis identified four factors that were significantly associated with the participants’ self-perceived dry mouth (Table 4). The odds of having self-perceived dry mouth among the participants living in SCWIs were 3.11 times (95 % CI 1.50–6.45) those of the elderly people in NHs. The odds of self-perceived dry mouth among participants who underwent regular oral examinations were 0.38 times (95 % CI 0.15–0.97) those of participants who did not undergo regular oral examinations. The odds of self-perceived dry mouth among participants wearing dentures were 2.62 times (95 % CI 1.28–5.34) those of individuals who did not wear dentures. The odds of self-perceived dry mouth among participants who could chew sticky foods reasonably well were 0.24 times (95 % CI 0.12–0.51) those of individuals with a poor ability to chew sticky foods.

**Table 4:** Factors associated with self-perceived dry mouth

Variable	Odds ratio (95 % CI)	*p* value
*Institutions type*		
Nursing home^a^	1	
Senior citizens welfare institutions	3.11 (1.50–6.45)	0.002
*Regular oral examinations*		
No	1	
Yes	0.38 (0.15–0.97)	0.040
*Denture wearing*		
No^a^	1	
Yes	2.62 (1.28–5.34)	0.008
*Ability to chew sticky foods*		
Poor^a^	1.000	
Good	0.24 (0.12–0.51)	0.000

*CI* confidence interval

^a^Reference group

## Discussion

This cross-sectional study examined factors affecting the self-perceived dry mouth of institutionalized older peoples. The results indicated that the type of LTC facility, which is an issue that has seldom been explored in previous studies, was one of the factors affecting self-perceived dry mouth. The results showed that the risk of dry mouth for residents of SCWIs was higher than that of residents of NHs. The caregivers’ provision of oral care in NHs was higher than that in SCWIs. NHs provide more medical care than SCWIs, and the links to relevant medical resources are more abundant [9]. As a result, it is necessary to develop early oral care strategies for elderly people in SCWIs.

This study determined the lack of regular oral examinations as one of the factors affecting dry mouth. As such, the role of regular oral examinations in the prevention and improvement of oral health cannot be denied. Most researchers on this topic have suggested the inclusion of regular oral examinations for coverage by medical insurance, as well as the provision of complete oral care and monitoring of oral problems caused by chronic diseases [22–24]. In Taiwan, oral examinations are covered by National Health Insurance (NHI). Therefore, dentists’ willingness to provide medical services in LTC facilities is low [9]. LTC facilities also lack adequate equipment and/or have no treatments areas [25], which further hampers the staff members’ ability to provide oral examinations to the older peoples’ at these facilities. Because oral examinations can reasonably reduce dry mouth, it is urgent for institutionalized oral health, medical plans to be developed.

The results from the current study showed that wearing dentures are one of the factors affecting self-perceived dry mouth, which was consistent with the results obtained by Campisi et al. (2008) [26]. Moreover, another study pointed out that the oral and denture cleaning habits of patients with dry mouth are generally poor, which exacerbates the discomfort associated with wearing dentures [27]. Kakudate et al. (2012) found a positive association between daily oral cleaning and dry mouth [8]. However, in the current study, oral health self-care behavior did not affect dry mouth. As such, it is important to conduct further studies to confirm the link between dry mouth and oral and denture cleaning among institutionalized older peoples.

The current study showed that the ability to chew sticky foods affected self-perceived dry mouth. Previous studies investigating the textures of food consumed by older people with dry mouth found that they typically decreased their intake of whole grains and increased their intake of fruits [28], suggesting that older people with dry mouth tend to be affected by hard or dry food textures. Their coping strategy is to avoid certain foods and choose alternatives. The results in the current study showed that sticky food textures affect dry mouth. Few studies have investigated the degree of stickiness that leads to chewing or swallowing difficulties among older people with dry mouth. Reilly et al. [29] used molecular gastronomy to enable control over food textures and the provision of food to people with different degrees of swallowing difficulty. Molecular gastronomy could be used in future studies to determine which degree of stickiness has an effect on chewing or swallowing among older people with dry mouth.

### Study limitations

This study had several limitations. First, drugs are an important factor affecting dry mouth. The face-to-face interviews with these study participants revealed that they were not aware of the drugs they were taking. As a result, this study could neither consider issues associated with the drugs taken by the participants nor determine the relationship between drugs and dry mouth. Future studies must take into account the drugs prescribed by physicians and pharmacists for LTC facility residents. Second, institutionalized older peoples are mainly those with physical weaknesses and disabilities. This study used face-to-face interviews to confirm the potential influences of oral health, self-care behavior, and oral health beliefs on institutionalized older peoples’ dry mouth; however, the sample size was small, which meant that the research results cannot be generalized to other contexts. Third, dry mouth is a subjective feeling. Therefore, this study used older peoples’ self-perceived dry mouth symptoms within the most recent 3 months in LTC facilities as the judgment criterion. Future cooperation with medical resources may help achieve an objective judgment. Moreover, the results of the Chinese version of the Summated Xerostomia Inventory (SXI), issued in 2013, showed that its reliability and validity in China are acceptable [30]. This inventory could be aggressively applied to Chinese regions in the future to understand the issues concerning Chinese older peoples’ self-perceived dry mouth.

## Conclusions

This study identified that the type of LTC facility, regular oral examinations, denture wearing, and the ability to chew sticky foods are factors affecting dry mouth. In terms of oral care strategies for use in the future, this study suggested providing oral care to improve and prevent dry mouth in institutionalized older people living in SCWIs who do not undergo regular oral examinations, wear dentures, or have difficulty chewing food.
